# Aging, Cellular Senescence, and Progressive Multiple Sclerosis

**DOI:** 10.3389/fncel.2020.00178

**Published:** 2020-06-30

**Authors:** Dimitrios Papadopoulos, Roberta Magliozzi, Dimos D. Mitsikostas, Vassilis G. Gorgoulis, Richard S. Nicholas

**Affiliations:** ^1^Molecular Carcinogenesis Group, Laboratory of Histology and Embryology, School of Health Sciences, National and Kapodistrian University of Athens, Athens, Greece; ^2^Department of Neuroscience, Biomedicine and Movement, University of Verona, Verona, Italy; ^3^First Department of Neurology, Aeginition Hospital, National and Kapodistrian University of Athens, Athens, Greece; ^4^Department of Neuroinflammation and Neurodegeneration, Faculty of Medicine, Imperial College London, London, United Kingdom; ^5^Department of Visual Neuroscience, Faculty of Brain Sciences, Institute of Ophthalmology, University College London, London, United Kingdom

**Keywords:** multiple sclerosis, cellular senescence, inflammation, remyelination, neurodegeneration, neuroprotection, senolytics

## Abstract

Aging is one of the most important risk factors for the development of several neurodegenerative diseases including progressive multiple sclerosis (MS). Cellular senescence (CS) is a key biological process underlying aging. Several stressors associated with aging and MS pathology, such as oxidative stress, mitochondrial dysfunction, cytokines and replicative exhaustion are known triggers of cellular senescence. Senescent cells exhibit stereotypical metabolic and functional changes, which include cell-cycle arrest and acquiring a pro-inflammatory phenotype secreting cytokines, growth factors, metalloproteinases and reactive oxygen species. They accumulate with aging and can convert neighboring cells to senescence in a paracrine manner. In MS, accelerated cellular senescence may drive disease progression by promoting chronic non-remitting inflammation, loss or altered immune, glial and neuronal function, failure of remyelination, impaired blood-brain barrier integrity and ultimately neurodegeneration. Here we discuss the evidence linking cellular senescence to the pathogenesis of MS and the putative role of senolytic and senomorphic agents as neuroprotective therapies in tackling disease progression.

## Introduction

Multiple sclerosis (MS) is a chronic, immune mediated disease of unknown etiology characterized by inflammatory demyelination, astrogliosis, neuronal and axonal loss involving the brain and spinal cord. The majority of MS patients follow an initial course with relapses and remissions (RR-MS) followed by a phase of progressive accumulation of disability termed secondary progressive (SP-MS). Ten to 15% of patients with primary progressive MS (PP-MS) exhibit gradual worsening from the start and typically PP-MS presents at an older age than RR-MS (Compston and Coles, 2008). The pathogenesis of the progressive disease courses (P-MS) is poorly understood. However, epidemiological evidence indicates that age is the strongest predictor for the transition from the relapsing phase, which is considered primarily inflammatory to the secondary progressive phase of the disease, which is mainly neurodegenerative (Trapp and Nave, 2008; Scalfari et al., 2011).

Although our understanding of the biological basis of aging remains incomplete, the prevailing hypothesis postulates that it is driven by the accumulation of irreparable molecular and cellular damage leading to an increased risk of functional decline, disease and ultimately death. Aging exhibits a great diversity of phenotypes and a loose connection between biological and chronological age, probably due to the stochastic nature of molecular damage and the complexity of the interaction between genetic and environmental factors (Kirkwood et al., 2005; Kirkwood and Melov, 2011). Aging is the most important risk factor for the development of neurodegenerative disease (Hou et al., 2019). Cellular senescence (CS) has been recognized as a key biological process underling normal aging (López-Otín et al., 2013; Gorgoulis et al., 2019) and evidence suggest that the accumulation of senescent cells with time may contribute to the pathogenesis of age-related and neurodegenerative disease (Kritsilis et al., 2018). Here, we review the data that support a role for cellular senescence in the pathogenesis of MS.

## The Senescence State

Several cell stressors have been identified as triggers of CS, which among others include oxidative stress, mitochondrial dysfunction, replicative stress, cytokines, irradiation, genotoxic agents presented in detail in Gorgoulis et al. (2019). Oxidative stress, mitochondrial dysfunction and cytokines, such as TGF-beta are key features of MS pathology (Gilgun-Sherki et al., 2004; Mahad et al., 2009; Haider et al., 2011; Elkjaer et al., 2019). Most of these triggering factors are associated with DNA damage and activate the signal transduction system of DNA damage response (DDR) (Nakamura et al., 2008). If the damage is irreparable DDR may elicit CS (Rodier and Campisi, 2011).

The senescent phenotype is typically associated with several metabolic and functional changes including stable cell cycle arrest, the expression of a senescence-associated secretory phenotype (SASP) and the accumulation of dysfunctional mitochondria (Coppé et al., 2010; Munoz-Espin and Serrano, 2014; Correia-Melo et al., 2016). SASP consists of pro-inflammatory cytokines, growth factors, cytotoxic mediators, metalloproteinases and reactive oxygen species (ROS). These are capable of affecting neighboring cells and converting them to senescence in a paracrine manner (Kuilman and Peeper, 2009; Acosta et al., 2013). Other changes characteristic of the senescent state are described in greater detail elsewhere (Munoz-Espin and Serrano, 2014; Gorgoulis et al., 2019). The senescence-associated changes reported specifically for CNS cells are summarized in Table 1.

**Table 1:** Observed hallmarks, features, and functional changes associated with CS in CNS cells.

**Cell type**	**Senescence-associated changes**	**SASP factors**	**Functional changes**	**References**
Neurons	γH2A.X and 53BP1 upregulation SAHF (macroH2A) SA-β-Gal activity↑ Lipofuscin accumulation	IL-6 protein	?	Sedelnikova et al., 2004; Jurk et al., 2012; Kritsilis et al., 2018
Astrocytes	Cell-cycle arrest SAHF (Hp1γ↑); 53BP1 foci↑ p16^*INK*4*A*^ mRNA and protein↑, p21 mRNA and protein↑, p53 mRNA↑ p38MAPK and NF-κB activation SA-β-Gal activity↑ EAAT-1 mRNA and protein↓, Kir4.1 mRNA and protein ↓, GFAP protein↓, S100β, TIMP-1 mRNA ↑; Lamin B1 mRNA↓	CXCL-1 mRNA TGF-β HMGB1 IL-6 mRNA and protein IL-8 mRNA and protein MMP-3 mRNA and protein MMP-9 mRNA	Loss of support of oligodendrocyte differentiation *in vitro* Loss of ability to support neuronal survival *in vitro*	Bitto et al., 2010; Salminen et al., 2011; Bhat et al., 2012; Al-Mashhadi et al., 2015; Görg et al., 2015; Nie et al., 2015; Crowe et al., 2016; Hou et al., 2017, 2018; Chinta et al., 2018; Turnquist et al., 2019; Limbad et al., 2020; Willis et al., 2020; Yabluchanskiy et al., 2020
Oligodendrocytes	γH2A.X upregulation SA-β-Gal upregulation	?	?	Al-Mashhadi et al., 2015
Microglia	Cell-cycle arrest Telomere attrition p38MAPK activation SA-β-Gal activity↑ SAHF	IL-6, IL-1β, TNF-α	Impaired phagocytic capacity in vitro^*^	Flanary and Streit, 2004; Flanary et al., 2007; Sierra et al., 2007; Bachstetter et al., 2011; Njie et al., 2012; Yu et al., 2012; Rawji et al., 2020
Oligodendrocyte progenitor cells (OPCs)	SA-β-Gal upregulation Increased DNA damage p21↑ and p16INK4A mRNA and protein↑ mTOR activation	?	Impaired proliferation and differentiation	Kujuro et al., 2010; Choi et al., 2016; Neumann et al., 2019; Zhang et al., 2019
Neural precursor cells (NPCs)	Flattened morphology Telomere attrition p16INK4A SA-β-Gal activity↑	HMGB1ROS production	Impaired adult neurogenesis *in vivo* Inhibition of oligodendrocyte differentiation *in vitro*	Ferron et al., 2004, 2019; Bose et al., 2010; He et al., 2013; Yang et al., 2017; Nicaise et al., 2019; Willis et al., 2020
Ependymal cells	p16^*INK*4*A*^ mRNA ↑ SA-b-Gal activity ↑	?	Impaired BBB function *in vitro*	Yamazaki et al., 2016; Yang et al., 2017
Pericytes	p16INK4A mRNA ↑ SA-β-Gal ↑activity	?	Impaired BBB function *in vitro*	Yamazaki et al., 2016

BBB, blood-brain barrier; 53BP1, p53 binding protein-1; CXCL-1, chemokine C-X-C motif ligand 1; CS, cellular senescence; GFAP, glial fibrillary acidic protein; H2A.X, H2A histone family member X; HMGB1, high mobility group box-1; Hp1γ, heterochromatin protein-1γ; IL, interleukin; MMP, metalloproteinase; mTOR, mammalian target of rapamycin; NPCs, neural progenitor cells; OLG, oligodendrocytes; OPCs, oligodendrocyte progenitor cells; p38MAPK, p38 mitogen-activated protein kinases; ROS, reactive oxygen species, SA-β-Gal, senescence-associated β-galactosidase; SAHF, senescence-associated heterochromatin formation; SASP, senescence-associated secretory phenotype; Timp-1, tissue inhibitor of metalloproteases-1; TNF-α, tumor necrosis factor-α.

* *Njie et al. (2012) and Rawji et al. (2020) have provided in vitro evidence of impaired phagocytic capacity of microglia from aged mice but have not provided evidence of CS*.

Although CS is a homeostatic response aiming to prevent the proliferation and neoplastic conversion of damaged cells (Munoz-Espin and Serrano, 2014) it also has a role in development (Rajagopalan and Long, 2012; Barbouti et al., 2019). Damaged senescent cells remain viable and metabolically active, they accumulate with aging and evidence suggests that their build-up may promote neurodegeneration (Rodier and Campisi, 2011). The detrimental effects of CS on the brain are due to the pro-inflammatory milieu formed by senescent cells that act as sources of inflammatory mediators (Coppé et al., 2010). CS-associated cell-cycle arrest may exhaust the regenerative capacities of adult progenitors, such as oligodendrocyte progenitor cells (OPCs) responsible for myelin repair. In addition, CS along with replication arrest is associated with extensive changes in gene expression, which indicate severe loss or alteration of physiological cell function (Purcell et al., 2014). Finally, endothelial cell senescence may compromise blood-brain barrier (BBB) integrity (Yamazaki et al., 2016), which is essential for preserving brain tissue homeostasis (Berthiaume et al., 2018).

## Cellular Senescence and Inflammatory Activity in MS

Both innate and adaptive components of the immune response are known to play key roles in the immunopathogenesis of MS (Weissert, 2013; Hemmer et al., 2015). Microglial cells, the resident representative of the innate immune response in the CNS (Ransohoff and Brown, 2012) are known to become senescent under specific circumstances. Cultured microglial cells can become senescent in response to chronic inflammatory stimulation by lipopolysaccharide treatment (Yu et al., 2012). Rat and human microglial cells from AD patients have been shown to undergo replicative senescence due to telomere shortening (Flanary and Streit, 2004; Flanary et al., 2007). With aging, microglial cells exhibit a dystrophic phenotype associated with functional changes, which seem to be distinct from the typical microglial reaction (Streit et al., 2004, 2009; Conde and Streit, 2006). Aged microglia exhibit decreased migratory and phagocytic capacity and secrete constitutively greater amounts of interleukin-6 (IL-6) and tumor necrosis factor-α (TNF-α) in culture (Njie et al., 2012; Rawji et al., 2020). The constitutive secretion of proinflammatory cytokines by microglia from aged mice is consistent with the SASP of senescent cells. Myelin clearance is a prerequisite for remyelination to occur (Kotter et al., 2001, 2006; Cantuti-Castelvetri et al., 2018) and impaired phagocytotic capacity of aged microglia and macrophages could hinder myelin repair in older patients. Nevertheless, the presence of senescent microglia and macrophages in MS and its models has not been shown.

With regard to the adaptive component of the immune response in MS, there is evidence of premature immunosenescence with T cell changes resembling those seen in the elderly (Thewissen et al., 2005). An expansion of CD4(+) CD28(–) T cells and a reduction of T-cell receptor excision circles (TREC) has been observed in the peripheral blood of MS patients (Thewissen et al., 2005). Peripheral blood CD4(+) CD28(-) T cells in patients and healthy controls exhibit an effector-memory T cell phenotype with cytotoxic properties, as they secrete cytotoxic granules in response to polyclonal stimuli and MS-related autoantigens. These CD4(+)CD28(-) T cells are attracted by increased levels of fractalkine and IL-15 and accumulate in demyelinated lesions (Broux et al., 2012, 2015). Similarly, a population of IgD(-)^−^CD27(-)CD11c (+)(CD21^low^) B cells, which have been associated with aging was demonstrated in higher proportions in the CSF and peripheral blood of MS patients than age-matched healthy controls. These IgD(-)^−^CD27(-)CD11c(+)(CD21^low^) B cells produced proinflammatory cytokines upon *ex vivo* stimulation and showed MHC class-II expression and costimulatory molecule expression capable to induce proinflammatory T cell responses. Their presence indicates that premature senescence of B cells may promote inflammation and thereby contribute to disease progression in MS (Claes et al., 2016).

Senescent neurons and glia accumulating in the MS brain and secreting SASP-related inflammatory mediators may represent an alternative source of inflammation independent of the immune cells that bring about innate and adaptive immune responses. We have previously provided evidence of senescent glial cells and neurons showing lipofuscin^+^ senescent glial cells in acute and chronic actively demyelinated white matter lesions from SP-MS cases using GL13 histochemistry (Kritsilis et al., 2018). All resident CNS cell types that under some circumstances acquire a senescent phenotype may become sources of parenchymal inflammation. The documented ability of senescent cells to convert neighboring cells to senescence via paracrine action of SASP mediators is consistent with the progressive nature of disability seen in P-MS (Acosta et al., 2013; Chen et al., 2015). Current therapeutic strategies fail to tackle disability progression in P-MS despite being efficacious in preventing MS relapses and new lesion formation thought to be mediated by adaptive immune responses (Pardo and Jones, 2017). This is consistent with the concept of resident glia and neurons secreting SASP-related factors that maintain a low-burning yet persistent and self-enhancing inflammatory environment not affected by immunomodulators and immunosuppressants. The glial cell types, which are prone to senescence in the MS lesions remain to be specified.

## Aging, Cellular Senescence, and Failure Of Myelin Repair

Endogenous myelin repair (remyelination) of axons surviving inflammatory demyelination is known to occur in MS (Patani et al., 2007). Remyelination contributes to restoration of impulse conduction along axons traversing demyelinating plaques and exerts a neuroprotective effect on remyelinated axons preventing axonal degeneration associated with chronic demyelination (Kornek et al., 2000; Franklin and Goldman, 2015; Mei et al., 2016). Adult oligodendrocyte progenitor cells (OPCs) comprising 5–10% of all CNS cells are primarily responsible for carrying out myelin repair following demyelinating events (Reynolds et al., 2002; Tripathi et al., 2010). The efficiency of myelin repair is known to gradually decline with time and it is shown to be least extensive in the SP-MS phase of the disease (Bramow et al., 2010). This suggests that age-related remyelination failure may contribute to disability progression seen at the advanced stages of MS (Kuhlmann et al., 2008; Goldschmidt et al., 2009). Aging has been shown to reduce the capacity for remyelination in several animal models of demyelination (Shen et al., 2008; Hampton et al., 2012; Cantuti-Castelvetri et al., 2018) in which remyelination inefficacy is associated with impaired recruitment of OPCs into demyelinated lesions and slower differentiation into myelinating oligodendrocytes (Sim et al., 2002). OPCs from aged rats show features of CS with increased levels of DNA damage, mitochondrial dysfunction and p38MAPK mRNA upregulation (Neumann et al., 2019). In addition, recent experimental evidence indicates that murine astrocytes aged in culture develop a pro-inflammatory senescence-like phenotype and lose their ability to support oligodendrocyte differentiation (Willis et al., 2020).

However, OPC senescence may not only be associated with aging. In experimental autoimmune encephalomyelitis (EAE) model of MS in young mice, OPCs exhibited cell-cycle arrest linked to an upregulation of sirtuin 1 (SIRT1) transcription, suggesting that failure of OPC proliferation may be due to CS (Prozorovski et al., 2019). Furthermore, *in vitro* exposure of cultured murine OPCs to Aβ oligomers triggered CS and inhibited myelin sheet formation indicating that toxic factors can elicit senescence in OPCs (Horiuchi et al., 2012; Zhang et al., 2019). In the APP/PS1 model of Alzheimer's disease OPCs expressing upregulated p16, p21, and senescence-associated-β-Galactosidase (SA-β-Gal) markers of CS have been identified in association with Aβ plaques and treatments aiming to remove senescent OPCs (senolytics) attenuated neuroinflammation and cognitive deficits, indicating that OPC SASP promotes neuroinflammation and functional impairment (Zhang et al., 2019).

Other progenitor cells including neural progenitor cells (NPCs) and mesenchymal stem cells (MSCs) also have a role in promoting remyelination and tissue repair (Nicaise et al., 2017; Rivera et al., 2019). It is documented that the adult CNS harbors multipotent neural progenitor cells (NPCs) that can produce neurons, astrocytes, and oligodendrocytes (Weiss et al., 1996; Johansson et al., 1999). They are thought to primarily reside in the subventricular zone (SVZ) and the subgranular zone (SGZ) of the dentate gyrus (Kriegstein and Alvarez-Buylla, 2009; Ming and Song, 2011). Most NPCs in the adult brain exist in a quiescent state (Ding et al., 2020) unless CNS injury or specific stimuli elicit their proliferation, migration and differentiation (neurogenesis) (Mothe and Tator, 2005). Evidence supports the functional significance of NPCs as hippocampal neurogenesis is critical for cognition (Suh et al., 2009; Christian et al., 2014) and its disruption is associated with cognitive impairment (Aimone et al., 2014). In addition, studies have demonstrated that adult NPCs from the subventricular zone (SVZ) and the spinal cord contribute to the generation of new oligodendrocytes and myelin repair in models of demyelination (Nait-Oumesmar et al., 1999; Danilov et al., 2006; Menn et al., 2006; Xing et al., 2014; Maeda et al., 2019). Thus, NPCs could provide an alternative source of myelinating oligodendrocytes and probably also a source of neurons in demyelinated MS lesions (Chang et al., 2008).

Aging is associated with progressive reduction in adult neurogenesis (Lugert et al., 2010; Cipriani et al., 2018), which is ascribed to a diminution of the pool of stem cells capable of activation and division (Lugert et al., 2010) and it is associated with functional impairment (Hollands et al., 2017). Accumulating evidence suggests that these NPCs are also prone to senescence. Cultured NPCs exhibit characteristics of senescence, such as enlarged and flattened morphology, increased levels of SA-β-Gal and p16 and decreased level of phospho-Retinoblastoma (pRb) upon long term incubation with Aβ oligomers (Ferron et al., 2004; He et al., 2013; Li et al., 2016). Cell cycle arrest of adult progenitor cells in the context of CS or inhibitory paracrine stimuli by neighboring senescent cells may impair progenitor proliferation, reduce the regenerative capacities of the CNS and render it susceptible to neurodegeneration. This notion is supported by *in vivo* evidence from the BUBR1 KO progeroid mouse model in which adult neurogenesis was impaired in the SGZ and SVZ in an age-dependent manner (Yang et al., 2017). In MS, SOX2+ NPCs from demyelinated white matter lesions of autopsy material and NPCs from induced pluripotent stem cell lines from P-MS patients were found to express markers of CS. These senescent progenitor cells exhibited impaired capacity to support oligodendrocyte maturation *in vitro*, compared to NPCs from age-matched controls. Proteomic and transcriptomic analysis of the P-MS NPC secretome identified high-mobility group box-1 (HMGB1) as a senescence-associated inhibitor of oligodendrocyte differentiation, which induces expression of epigenetic regulators. HMGB1 was found to be expressed by progenitor cells in MS white matter lesions (Nicaise et al., 2019). Failure of spontaneous remyelination in MS may be at least partly due to conversion of OPCs and other neural progenitor cells to a state of CS induced by MS-specific triggers including oxidative stress, chronic inflammation, mitochondrial dysfunction and aging.

## Neurodegeneration and Disability Progression in Ms

Neuroaxonal loss is the pathological correlate of irreversible disability (Trapp et al., 1998; Papadopoulos et al., 2006). Although axonal loss can be an early feature of MS pathology associated with inflammatory lesion formation, in P-MS new focal inflammatory demyelinating plaques are rare (Trapp and Nave, 2008). Neuroaxonal loss in P-MS is driven by neurodegenerative mechanisms, which are poorly understood (Lassmann et al., 2007, 2012; Trapp and Nave, 2008).

Recently, telomere length of white blood cells (WBCs) used as a measure of WBC biological age was found decreased in P-MS patients compared to aged-matched controls (Habib et al., 2020). Moreover, shorter telomere lengths correlated with greater brain atrophy and higher levels of disability (Krysko et al., 2019), suggesting that biological aging contributes to neuroaxonal loss and disability progression in MS. Total brain atrophy, as assessed by MRI, reflects primarily gray matter atrophy due to neurodegeneration (Filippi et al., 2012) and correlates with long-term disability in MS (Fisniku et al., 2008; Filippi et al., 2013). Combined longitudinal MRI-based brain morphometry and brain age estimation using machine learning, revealed accelerated progressive brain aging in MS patients compared to healthy controls, which was related to brain atrophy and increased white matter lesion load (Høgestøl et al., 2019; Cole et al., 2020). Healthy aging is associated with brain cell loss, which may account up to 0.4% of brain volume per year (De Stefano et al., 2016). Both apoptotic and senescent cells are cleared by the immune system in a highly regulated manner and may contribute to age-related brain volume loss (Hoenicke and Zender, 2012; Ovadya et al., 2018).

Although post-mitotic cells do not fit the strict definition of CS, there is evidence of neurons developing a senescence-like phenotype. Neurons of aged mice have been shown to accumulate hallmarks of cellular senescence including double-strand DNA breaks, heterochromatinization, upregulation of SA-β- Gal, p38MAPK activation and production of SASP-related mediators including ROS and IL-6 (Sedelnikova et al., 2004; Jurk et al., 2012). The demonstration of neuronal granular cytoplasmic lipofuscin deposits in subpial demyelinated cortical lesions and normal appearing cortex from SP-MS cases using GL13 histochemistry supports the notion that human neurons may also acquire a senescence-like phenotype in MS (Kritsilis et al., 2018).

Although the functional state of senescent cells has not been fully elucidated, CS is accompanied by changes in gene expression and phenotypic changes, which constitute serious restrictions in the functionality of cells (Purcell et al., 2014). The number of senescent cells increases with age (Rodier and Campisi, 2011). When the number of dysfunctional senescent cells exceeds a certain threshold in a brain with reduced reserves due to age and MS-related cell loss, brain tissue function is likely to become compromised (Oost et al., 2018).

Neuronal survival strongly depends on the functional integrity of glial cells. Cultured astrocytes from aging rats have been found to upregulate the activity of SA-β-Gal, a marker of CS while they showed a reduced ability to maintain survival of co-cultured neurons, thus associating astrocyte senescence with neurodegeneration (Pertusa et al., 2007). Senescent astrocytes expressing P16^INK4A^ and secreting metalloproteinase-1 (MMP-1) have been found in post-mortem tissues of Alzheimer's disease patients (Bhat et al., 2012). Primary human astrocytes made senescent by X-irradiation were found to downregulate genes encoding glutamate and potassium transporters leading to neuronal death in co-culture assays. These findings indicate that excitotoxicity, a recognized mechanism of neurodegeneration in MS (Werner et al., 2001) may result from impaired homeostatic capacities of senescent astrocytes (Limbad et al., 2020). Notably, dysregulated splicing of several genes from human senescent astrocytes has been demonstrated and an association between peripheral blood GFAPa, TAU3 and p14ARF isoform levels and cognitive decline has been demonstrated, indicating a link between astrocyte senescence and disability (Lye et al., 2019). Although no evidence of astrocyte senescence has been shown in association with MS pathology, their key role in neuron-glial crosstalk, regulation of neuronal metabolic and ion homeostasis and modulation of synaptic transmission via glutamate suggest that age-related astrocytic senescence may promote neuronal dysfunction and degeneration, contributing to MS progression.

Age-related accumulation of senescent endothelial cells is linked to impaired tight junction structure and compromised blood-brain barrier (BBB) function (Farrall and Wardlaw, 2009; Yamazaki et al., 2016; Castellazzi et al., 2020). Several lines of evidence from human studies and experimental animal models support a key role for fibrinogen in neuroinflammation (Davalos and Akassoglou, 2012). Blood-derived fibrinogen has been shown *in vivo* to interact with microglia via the CD11b/CD18 integrin receptor leading to perivascular microglial activation and axonal loss (Davalos et al., 2012). Fibrinogen has been found at the edge of chronic active lesions, which exhibit ongoing inflammatory demyelination, but not in inactive lesions, suggesting that fibrinogen may play a role in sustained inflammation even in the chronic setting. Endothelial senescence leading to a constantly leaky BBB may permit fibrinogen to diffuse into the brain parenchyma and drive axonal damage and loss mediated by persistent microglia activation as well as inhibition of remyelination (Petersen et al., 2018). The sustained nature of age-related BBB leakiness is consistent with the putative role of CS in neuroaxonal loss-mediated disability progression in MS.

## Can Senolysis be Neuroprotective?

Currently, there is an unmet need for neuroprotective treatments that can effectively prevent disability progression in MS. A growing body of evidence implicates CS in the pathogenesis of neurodegeneration in a number of settings (Martínez-Cué and Rueda, 2020), rendering CS a promising target for neuroprotection. Anti-senescent or senotherapeutic approaches may involve the selective death of senescent cells (senolysis) to reduce the load of senescent cells and their detrimental effects on tissues. Alternatively, senotherapy may be based on the modulation of the senescent cell phenotype (senomorphism) in order to block the damaging effects of the SASP or other senescence-associated mediators (Kirkland et al., 2017). Senotherapy in MS would aim at preventing senescence-associated chronic inflammation, loss of cell function and neuroaxonal loss and promoting remyelination. Compounds with senolytic or senomorphic actions have been studied *in vitro* and *in vivo* with promising results (Myrianthopoulos et al., 2019; Thoppil and Riabowol, 2020). Evidence from genetically modified mice support the neuroprotective potential of senolytic manipulation. Lifelong elimination of p16INK4A cells in BubR1 progeroid mice by activation of an INK-ATTAC transgene substantially delayed age-related disease, whereas late life elimination of p16INK4A cells attenuated these age-related pathologies (Baker et al., 2011). In addition, a senolytic compound (ABT263) was shown to attenuate tau phosphorylation and aggregation and to improve memory deficits in the PS19 transgenic model of tau-dependent neurodegeneration, by removing senescent glial cells (Bussian et al., 2018).

Fasting and metformin treatment could reverse the senescent state of rat OPCs and improve remyelination capacity (Neumann et al., 2019). Furthermore, rapamycin treatment modified the senescent state of progressive MS patient-derived NPCs produced from induced pluripotent stem cells and improved their capacity to promote OPC differentiation *in vitro*, providing evidence that senomorphic treatment can promote remyelination in MS (Nicaise et al., 2019). Simvastatin has shown efficacy in delaying brain atrophy and disability progression in MS trials (Chataway et al., 2014). This neuroprotective effect may be mediated via its senomorphic actions, which include downregulation of p38MAPK activation, SASP markers, TNFa, and GM-CSF as shown in other settings (Liu et al., 2015; Ayad et al., 2018). Many promising compounds with senolytic or senomorphic activity, such as metformin or simvastatin used with different indications could be repurposed and used as neuroprotectants combined with currently available immunomodulators. Immune-mediated physiological clearance of senescent cells could potentially be therapeutically enhanced by medications or vaccines aimed at priming the immune response to remove specific senescent populations (Burton and Stolzing, 2018; Song et al., 2020). Reprogramming of senescent cells may be another approach (Tamanini et al., 2018; Mahmoudi et al., 2019).

On the other hand, treatments aiming at the disease processes that precede and accelerate CS, such as inflammation, oxidative stress and mitochondrial dysfunction at the earliest stages of the disease may delay CS and hinder CS-related neurodegeneration. Furthermore, senescence-inducing practices and medications including exposure to ionizing radiation and DNA-damaging chemotherapeutics should be avoided. Interestingly, approved MS treatments, such as corticosteroids, beta-interferons and mitoxantrone should be re-evaluated for their long-term effects given that they have been shown to promote CS (Moiseeva et al., 2006; Ikeda et al., 2010; Poulsen et al., 2014).

## Concluding Remarks

**Figure 1:**
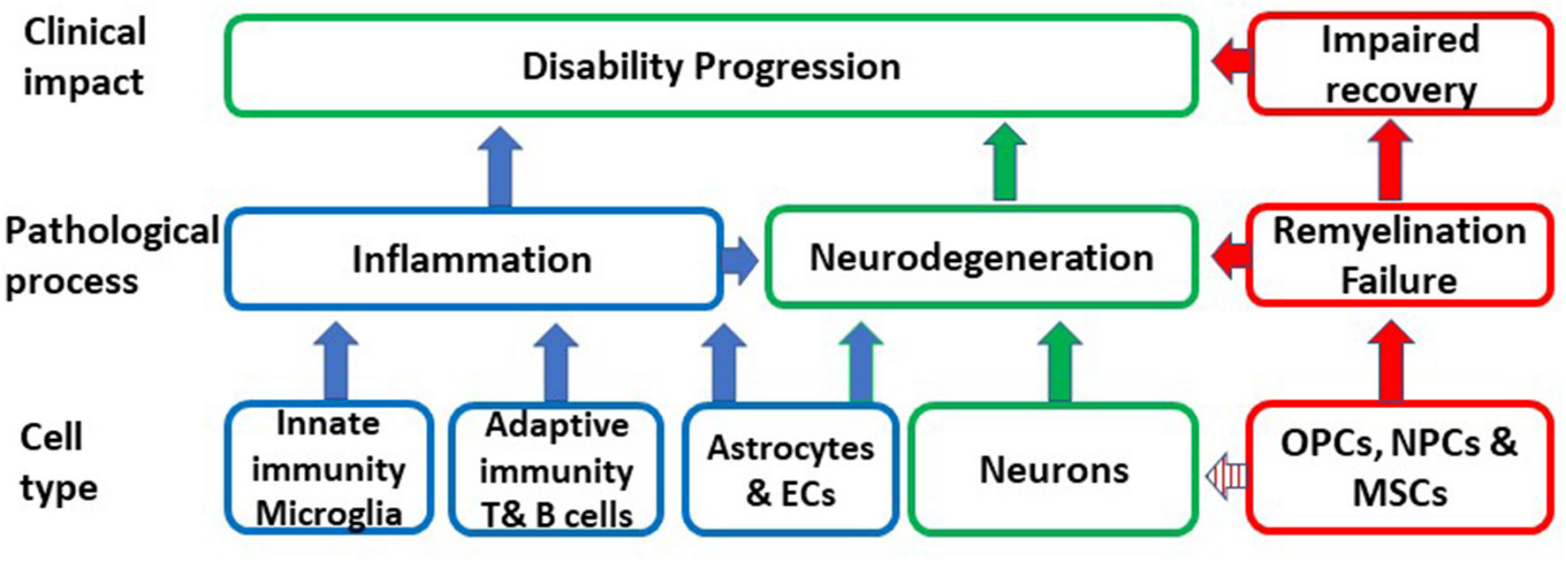
Schematic representation of the putative impact of the conversion of different cell types to senescence on inflammation, remyelination, neurodegeneration and ultimately on disability progression. OPCs, oligodendrocyte progenitors; MSCs, mesenchymal stem cells; NPCs, neural progenitor cells; EC, endothelial cells.

Aging is an important risk factor for the development of several neurodegenerative diseases including P-MS, where the neurodegenerative component dominates. A primary causative role of CS in MS is highly unlikely given the great diversity which characterizes aging-related neurodegenerative pathologies that have CS as a common feature. However, CS may be a shared mechanism, which substantially contributes to the pathogenesis and impact of neurodegenerative diseases and thereby may determine disease susceptibility, age at disease presentation and rate of progression. In MS, senescence may be responsible for chronic non-remitting inflammation, which is not amenable to immunomodulation, lost or altered glial and neuronal function, failure of remyelination, impaired BBB integrity and neurodegeneration (Figure 1).

Nevertheless, current evidence for a role of CS in disability progression in MS is intriguing but limited and indirect. Shedding light on CS and its role in neurodegeneration is essential to safely exploit it therapeutically. To facilitate these efforts a thorough histopathological investigation of post-mortem MS tissue at various disease stages and levels of disability would inform us of the extent, timing, particular cell types converted to senescence and all features of pathology associated with the accumulation of senescent cells. A more concise understanding of the biology of CS of neural cells, its triggers and mediators is required. Advanced human 3D and organoid culture techniques (Marton et al., 2019; Yoon et al., 2019) could help identify the specific factors that induce CS and the contribution of each cell type to tissue injury. Transgenic animal models of demyelination could provide mechanistic evidence to disentangle the detrimental effects of senescent cell types and their mediators and become platforms on which to test senotherapeutic agents. The complex physiological and pathophysiological roles of CS, along with the cell-type specific variability in senescence triggers and phenotypes, necessitates a cautious approach to avoid pitfalls when dealing with such a multifaceted biological process. If senescent cells are proven to drive neurodegeneration senotherapy may become the groundbreaking neuroprotective strategy to prevent and potentially reverse progressive disability in MS.

## Author Contributions

Literature search by DP, RM, DM, and RN. The first draft of the manuscript was written by DP and RN. RM, DM, and VG critically revised the manuscript. All authors contributed to the article and approved the submitted version.

## Conflict of Interest

The authors declare that the research was conducted in the absence of any commercial or financial relationships that could be construed as a potential conflict of interest.
